# Small-Molecule Binding Aptamers: Selection Strategies, Characterization, and Applications

**DOI:** 10.3389/fchem.2016.00014

**Published:** 2016-05-10

**Authors:** Annamaria Ruscito, Maria C. DeRosa

**Affiliations:** Department of Chemistry, Carleton UniversityOttawa, ON, Canada

**Keywords:** aptamer, small molecule, SELEX, *in vitro* selection, biosensor

## Abstract

Aptamers are single-stranded, synthetic oligonucleotides that fold into 3-dimensional shapes capable of binding non-covalently with high affinity and specificity to a target molecule. They are generated via an *in vitro* process known as the Systematic Evolution of Ligands by EXponential enrichment, from which candidates are screened and characterized, and then used in various applications. These applications range from therapeutic uses to biosensors for target detection. Aptamers for small molecule targets such as toxins, antibiotics, molecular markers, drugs, and heavy metals will be the focus of this review. Their accurate detection is needed for the protection and wellbeing of humans and animals. However, the small molecular weights of these targets, including the drastic size difference between the target and the oligonucleotides, make it challenging to select, characterize, and apply aptamers for their detection. Thus, recent (since 2012) notable advances in small molecule aptamers, which have overcome some of these challenges, are presented here, while defining challenges that still exist are discussed.

## Introduction

**Figure 1:**
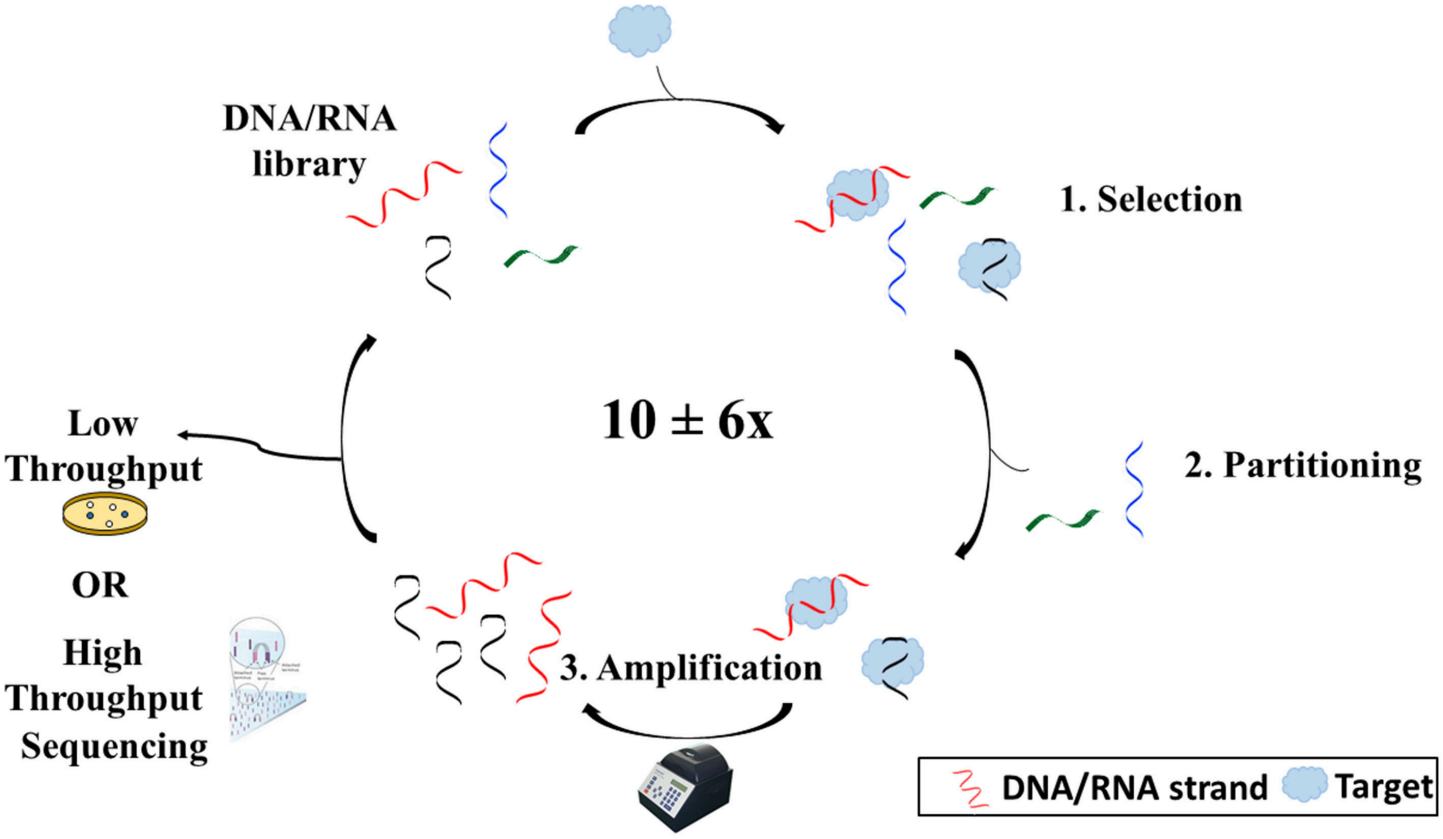
**Overview of the SELEX process**. SELEX begins with an oligonucleotide library consisting of ~10^16^ different sequences. The library is then incubated with the target (1). Some sequences will bind to the target and others will not. Sequences that have bound to the target are then separated from those that have not (2). After separating the sequences from the target, the sequences are copied and amplified using polymerase chain reaction (3). Sequences are then reintroduced into the process several times before being sequenced via either high throughput or low throughput sequencing.

While DNA is generally known as the biological macromolecule responsible for the storage of hereditary information, it can also act as an affinity probe or molecular recognition element for a variety of applications from therapeutics to biosensing. A DNA molecule which functions as such is termed an aptamer. Aptamers are single-stranded, synthetic oligonucleotides (DNA or RNA) which fold into 3-dimensional shapes capable of binding non-covalently and with high affinity to a target molecule. They can bind with such specificity that they can differentiate enantiomers and molecules that differ by as little as one functional group. Aptamers are generated via an *in vitro* process known as the Systematic Evolution of Ligands by EXponential enrichment, also known as SELEX. Tuerk and Gold (1990), aiming to find an RNA aptamer sequence that would bind T4 DNA polymerase, first termed the process. Many variations were made to the original SELEX process to better suit the needs of the researcher. Overall, however, SELEX is made up of three main steps: selection, partitioning, and amplification (Figure 1).

The selection step involves the incubation of an oligonucleotide library with a target molecule. The library generally consists of up to 10^16^ different sequences (Ellington and Szostak, 1990; Tuerk and Gold, 1990) each containing a random region flanked by two known sequence regions. The library can be tagged (radiolabel, fluorophore, etc.) to allow for monitoring, although depending on the SELEX method, this is not always necessary. The length of the random region varies based on a balance of three parameters: size of target, cost, and diversity. While a recent analysis of all reported selection experiments from 1990 to 2013 showed no statistically significant correlation between template length and affinity when all target types were examined together (McKeague et al., 2015b), it has been suggested that it may be better to have a library of short sequences for smaller molecules to try to close the size gap between the aptamer and small molecule, such as to limit crowding and increase selection efficiency (Neves et al., 2015). Also, the longer the sequence, the harder and more expensive it will be to synthesize. However, the longer the random region, the more possible conformations that the aptamer can adopt and the better the chance of finding an aptamer for the target (Nieuwlandt, 2000).

The partitioning phase involves the separation of sequences that have bound to the target from those that did not bind. There are numerous methods for performing this step, depending on the type of SELEX chosen. These methods are essentially mass-based or wash-based. Mass-based methods, such as size-exclusion filtration, rely on a distinguishable difference in mass of the aptamer upon complex formation that can be exploited for partitioning (Ozer et al., 2014). Wash-based methods require the immobilization of either the target or the oligonucleotide library, such that washes with solution would separate sequences that bound from those that did not.

Finally, amplification involves making an abundance of copies of the sequences that have bound to the target using polymerase chain reaction. The known flanking regions on each sequence make up the primer-binding sites to allow amplification. Since so few library sequences are recovered following partitioning, copies are required to make up the enriched library for continuation to the next round.

This three-step process is usually repeated several times with continuously changing conditions to allow for increased stringency. Only sequences with high affinity to the target would be able to bind in more stringent conditions. However, it is recommended to increase stringency after the first few rounds. Initially, the oligonucleotide pool is quite diverse, with many sequences in low frequency, which run the risk of being lost in the early rounds.

Aptamers can be used in a wide range of applications due to their unique properties. As such, fields such as medicine, cell and microbiology, and analytical chemistry are interested in aptamers for their potential use in drug development, cell tracking and viral and bacterial detection, and chromatographic separation, respectively (Sefah et al., 2009). In particular, aptamer-based biosensors and assays are being investigated as possible alternatives to conventional antibody-based technology. Aptamers are highly comparable to antibodies, in that they both bind to their targets with high affinity and selectivity. Nowadays, antibodies are the gold standard for use in biosensors or as drug components, however, aptamers have been increasing in popularity since their introduction in the early 1990s due to their advantageous properties. Unlike antibodies, aptamers are synthesized *in vitro*, which is not only a more ethical approach than using animals or cell cultures but also substantially less expensive and with little-to-no discrepancies between batches (Smith et al., 2007). In addition, aptamers have much longer shelf life than antibodies and are stable over a wide range of conditions; for example, while aptamers can renature following exposure to high temperatures, antibodies are irreversibly denatured. By exploiting these properties, one can select for aptamers in the conditions under which the aptamer would eventually be used, a unique benefit. Furthermore, due to the *in vitro* process aptamers can be selected for molecules that do not elicit an immune response; a circumstance which is necessary for antibody production. However, drawbacks to aptamers do exist. Aptamers have an overall negative charge making them hydrophilic, and are rapidly degraded by nucleases. With the exception of modified aptamers such as SOMAmers (Slow Off-rate Modified Aptamers; Rohloff et al., 2014), aptamers only contain 4 nucleic acid building blocks compared to the 22 amino acid building blocks of proteins, limiting their diversity of possible secondary and tertiary structures. Some drawbacks can be overcome, such as modifying the aptamer backbone or adding particular functional groups to increase their stability (Mayer, 2009; Tolle and Mayer, 2013). However, while SOMAmers and other modified aptamers offer more diversity and stability, custom synthesis adds to the costs.

Recently there has been an increase in demand for the detection of small molecules (<900.0 g/mol). These molecules include toxins, antibiotics, molecular markers, drugs, heavy metals, and ions. Industries such as pharmaceutical/medical require the quick and sensitive detection of small molecules such as molecular markers of disease, drugs, or metal ions for monitoring of human and animal health. The agriculture industry requires measuring levels of mycotoxins and heavy metals, among others, to ensure food safety. The detection of drugs is of great importance for forensic analysis as well. Because there is increasing need for small molecule detection, there must be a detection method that is sensitive, reliable, affordable, ideally portable, and simple to use. Aptamer-based sensors are becoming a promising alternative to conventional methods for small molecule detection. However, compared to the number of aptamers for larger target molecules (proteins, cells, etc.) reported in the literature, there are very few for small molecules. Only 25% of all reported aptamer selections from 1990 to 2013 were for small molecule targets (McKeague et al., 2015b). As will be discussed in upcoming sections, this discrepancy can be attributed in large part to technical challenges that affect selection and characterization.

This review exposes the challenges associated with small molecule aptamer selection, characterization, and aptamer-based detection systems. This topic has been reviewed previously (McKeague and DeRosa, 2012) and this review will serve as a follow up which will focus on the highlights and effort put forth by the research community since 2012 to overcome several of the challenges in each stage of the aptamer process to increase efficiency and likelihood of success. Highlights include new methods for selecting small molecule aptamers, a workflow comprising the methods and assays for aptamer screening, characterization, and functional verification, methods for aptamer truncation, and also the application of aptamers into various types of sensors which were previously difficult or unsuitable for small molecule aptamers.

## Selection (SELEX) of small-molecule binders

An examination of published selection data from 1990 to 2013 confirmed that aptamers selected for small molecule targets have lower affinity than those selected for other targets, on average (McKeague et al., 2015b). While the reasons for the poorer performance of small molecule aptamers remain to be elucidated, several technical challenges associated with the SELEX experiment are thought to play a role. One of the main challenges of performing SELEX with a small molecule target is the partitioning of bound and unbound sequences. The small size of the target does not lead to extensive mass difference between unbound aptamer and bound complex, complicating the separation process. As a result, small molecule selections typically involves immobilization of one of the biomolecules. Although there are a variety of current SELEX methods suited for small molecules (McKeague and DeRosa, 2012), they mostly stem from two main types, namely conventional/Flumag and Capture/structure-switching. Conventional SELEX was first performed by Ellington and Szostak (1990) for the selection of aptamers against organic dyes. This process involves the conjugation of the target to a solid support matrix, sepharose for example. Sequences that bind the target were separated from those that do not bind with multiple washes, then the sequences were subsequently eluted from the beads and amplified using polymerase chain reaction for the next round. With this method, the oligonucleotide pool is unmodified and free in solution, which eliminates complications involved in conjugating the pool to the bead. On the other hand, sepharose is not an ideal solid support as a large concentration of target is required for loading. Flumag SELEX was first performed by Stoltenburg et al. (2005) as proof-of-concept for the selection of aptamers against the protein streptavidin. What defines this method is the conjugation of the target on magnetic beads, which allows for more rapid selections. Relative to agarose and sepharose beads, magnetic beads are much more expensive, yet much less target is required to load the magnetic beads. The lower the amount of target, the more competition there is amongst the sequences in the pool, resulting in the selection of high affinity binders (Nieuwlandt, 2000; Stoltenburg et al., 2005). However, by immobilizing the target, the selection is being performed on a conjugate rather than the target alone. Under these circumstances, non-specific binding may take place, and the affinity may be negatively affected in future applications if target is free in solution.

From the two aforementioned SELEX methods, many more methods were developed. An often-used method is Structure-Switching or Capture SELEX; structure-switching SELEX was invented by Nutiu and Li (2003) for the selection of aptamers against the four standard NTPs. It is the opposite of Flumag SELEX with regards to target immobilization, and relies on a conformational change in the aptamer sequence upon target binding, coined as “structure-switching.” Rather than immobilizing the target to the solid support matrix of choice, a fluorescent oligonucleotide pool is tethered and the target is free in solution. To tether the pool to the matrix, a docking sequence complementary to a known region on every strand is conjugated to the matrix. The strand is bound to the docking sequence and the pool-labeled beads are exposed to the target. Only the sequence that changes structure conformation upon binding to the target will effectively release itself from the dock. Sequences found in solution following incubation with target are sequestered and amplified in preparation for the next round. Capture SELEX is ideal for molecules that are difficult to immobilize, such as small molecules. In addition, it provides high detection specificity (Stoltenburg et al., 2012); in order to overcome the attraction to the docking sequence, there must exist a sizeable change in the secondary and tertiary structure of the oligonucleotide upon target interaction. As will be discussed in future sections, this structural change can be exploited, as many characterization and sensor development methods rely on structural changes upon binding to target to be effective. However, tethering the oligonucleotide pool may have repercussions after SELEX if the potential aptamers are to be used free in solution in the downstream application, as the change in shape and conformation might not be the same, thus affecting the binding affinity of the aptamer.

Both conventional and capture SELEX have been used to select small-molecule binding aptamers successfully. However, in an attempt to overcome various complications associated with small molecule SELEX, the following advances have recently been made.

To address the large size difference between the small molecule and the aptamer, Handy et al. (2013) made use of a hapten-protein carrier bound to the target of interest, saxitoxin (M_W_: 299.3 g/mol). Saxitoxin is a marine toxin responsible for human syndrome paralytic shellfish poisoning. This concept was previously employed in an attempt to increase the immunogenicity of saxitoxin for the production of anti-saxitoxin antibodies. The already-established reaction for target immobilization involves the protein carrier keyhole limpet hemocyanin which is reacted to the target via a linker, Jeffamine. Keyhole limpet hemocyanin is subsequently conjugated to epoxy functionalized magnetic beads via the amine end. The need for protection of other functional groups was not required, thus simplifying the surface chemistry involved. By conjugating saxitoxin to the protein, saxitoxin is essentially converted to a large molecule, thereby closing the gap in the size difference between saxitoxin and the sequences, and increasing the chances of finding an aptamer. Another advantage to using a protein is the ability to evaluate the conjugation efficacy of the protein-target complex to the bead before selection. In this case, the beads with and without the protein-target complex were subjected to endoprotease trypsin, and the resulting peptides were analyzed using LC-MS/MS. This method confirmed successful conjugation to the beads. Conjugating the target directly onto the beads is possible but requires an alternative method for confirming conjugation, such tracking the target with a label or with their inherent fluorescence or inherent absorption in the UV-Visible spectrum.

This method successfully selected an aptamer for saxitoxin, with the aptamer binding in a concentration-dependent manner. Since the small molecule was essentially converted to a larger one, the protein addition was a relatively inexpensive solution to the size difference problem of small molecules and sequences. The drawback of this method, however, is that aptamers are selected for a conjugate rather than a small molecule alone. Nevertheless, the involvement of the protein proved to be valuable by easing conjugation and allowing for coupling evaluation.

**Figure 2:**
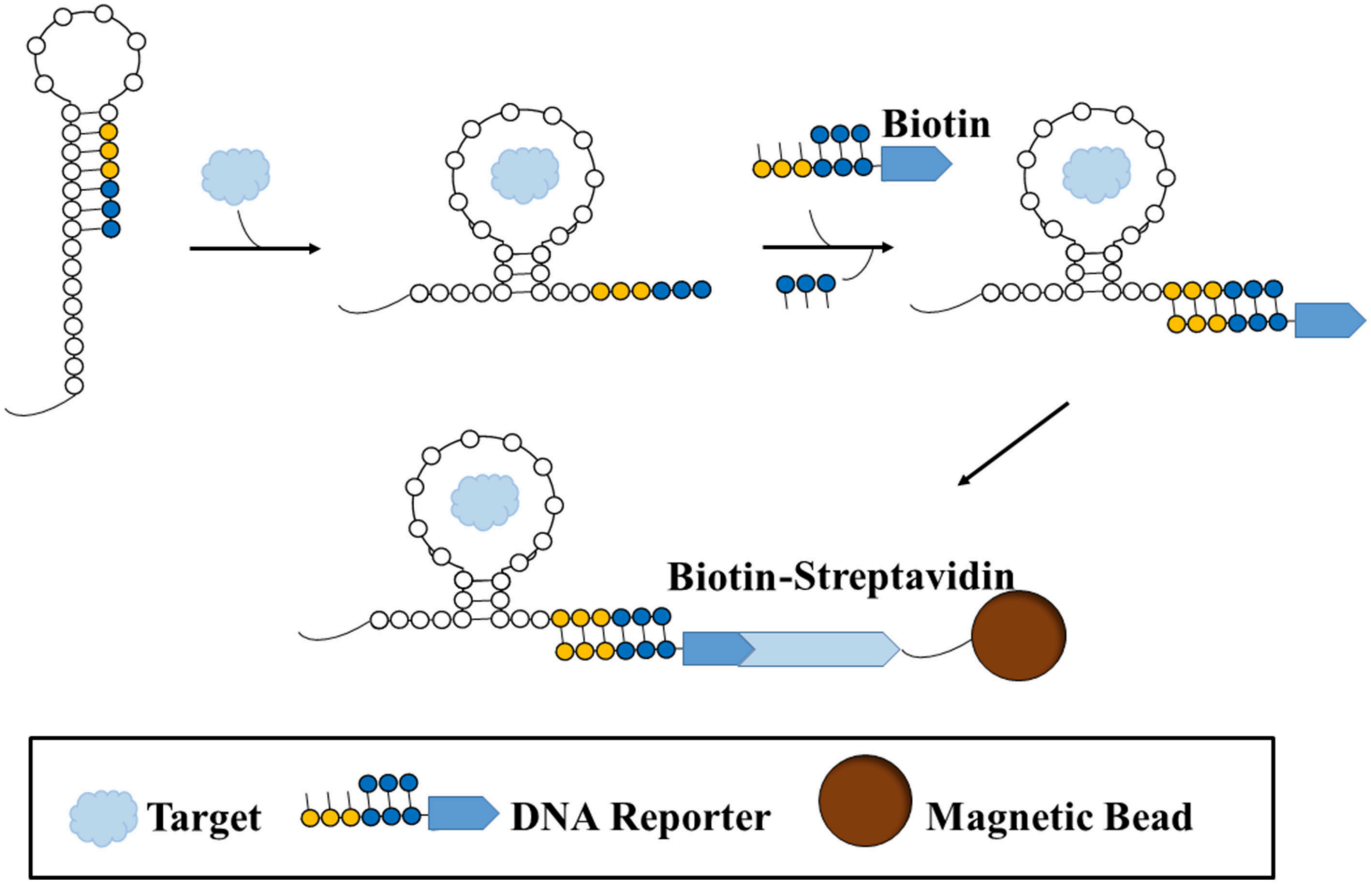
**A type of structure-switching SELEX for aptamer selection**. A sequence is folded in such a way that a binding site (yellow-blue) is hidden. Once target is introduced and bound to the sequence, the sequence undergoes a structural shift that exposes the binding site, such that a biotin-labeled double-stranded DNA reporter is free to bind to the site. Since the reporter preferentially binds to the binding site on the sequence, the shorter strand on the reporter becomes displaced. Thus, the sequence that binds the target and results in a structural shift becomes labeled with a biotin reporter. The sequence is then sequestered and separated from non-binding or non-shifting sequence structures with a streptavidin-conjugated magnetic bead.

Another promising variation of small molecule SELEX was performed by Martini et al. (2015). In order to evade the issues surrounding target or oligonucleotide pool immobilization, this variation of small molecule SELEX involves the use of an RNA library based on the structure of a riboswitch. Riboswitches are aptamers found in nature that change conformation upon binding to a ligand, thereby exposing a sequence in which a protein could bind and initiate a biochemical process. The team Martini et al. utilized a synthetic library version of a variant riboswitch for thiamine pyrophosphate (TPP, M_W_: 425.0 g/mol), a coenzyme and essential vitamin. This library was modified from the original riboswitch, such that four regions responsible for ribozyme activity were randomized, and the RNA pool and TPP are free in solution. Upon binding of a sequence to the target, a change in conformation of the sequence is exhibited, exposing an area for a short double stranded biotin-labeled DNA reporter with a complementary sequence to bind (Figure 2). Upon binding to the RNA sequence, a strand on the reported is displaced. Sequences which have bound to the reporter are isolated with streptavidin beads whereas those that do not change conformation cannot bind the biotin-labeled reporter and thus are discarded. Using this approach, only three rounds were required to obtain a high affinity RNA aptamer for TPP. The pools were sequenced and strand displacement was monitored with a fluorophore-quencher reporter (in place of the biotin-labeled reporter) after every round to monitor enrichment and activity, respectively.

Sequence diversity was decreased after the first selection round whereas displacement activity increased. In fact, the sequence which represented a large part of the pool in the final round had the highest level of displacement activity. In this situation, due to the exposure of previously obstructed sequence upon target binding, free equilibrium binding can take place between the strands and the target, followed by the capture and immobilization onto a bead only after the binding has taken place. Therefore, the usual complications involved in immobilizing the target or the pool such as non-specific binding, complicated surface chemistry, the possible change in affinity to target when not immobilized, are no longer of concern. In addition, after SELEX is terminated and multiple sequences are chosen for further testing, this can be further adapted as a screening method for characterizing potential aptamers and ultimately as a biosensor.

Quite commonly, 10 ± 6 (McKeague et al., 2015b) SELEX rounds are performed before the process is stopped and high affinity binders are screened. The lack of direct affinity and specificity monitoring after each round prevents one from knowing if and when the pool is being enriched, as well as if the sequences in the pool have high specificity to the target. Good binders may appear earlier than thought possible and thus costs and time expended for extra rounds would be futile, particularly if high affinity and specificity binders are not obtained in the end. Thus, in an effort to determine if selections with small molecules can be terminated early in the process, Spiga et al. (2015) performed capture SELEX in combination with SPR and Illumina high throughput sequencing to develop an aptamer for the antibiotic tobramycin (M_W_: 467.5 g/mol). Capture SELEX for small molecules can be time consuming and arduous due to the many rounds that must be performed. To determine when and which high affinity binders emerge at high frequency in the pool, SPR and high throughput sequencing were performed following each round of SELEX in order to assess binding to the target and for sequencing for enrichment analysis, respectively. The high throughput sequencing allows for quantitative determination of each sequence in the pool and also its enrichment relative to previous rounds. By using SPR and sequencing, the binding and pool enrichment for the target was directly monitored without waiting until the end of SELEX. By doing so at every round, a clear understanding of how well the pool is responding to the target was provided, such that SELEX could be ended when enrichment peaks and when there is sufficient binding to the target. In most cases, enrichment is determined indirectly based on how much of the library is eluted compared to a control. This approach is not always accurate as there is no measure of affinity of the pool to target or pool complexity. Ending SELEX prematurely results in a pool that is insufficiently enriched, thus the best aptamers may not be found. Alternatively, if too many rounds are performed and conditions are too stringent, high affinity aptamers may be lost and never recovered. Also, more rounds are associated with frequent pool amplifications that can skew the pool due to polymerase chain reaction bias. The researchers of this study found sequence convergence and maximal binding at round 12, however sequence families specific for the target began to emerge in round 8 where binding was observed. In addition, the enriched sequences which made up the bulk of the pool in the last round (round 12) were found to be the most enriched of much earlier rounds. In the end, high affinity aptamers for tobramycin that were comparable to existing tobramycin aptamers were obtained. Although the sequencing can be quite costly, it is highly likely that the cost will only decrease as this method becomes more common.

Another challenge associated with the selection of aptamers for small molecules is the lack of epitopes or functional groups available for strong aptamer binding. In the case of monosaccharides glucose, fructose, and galactose (each M_W_: 180.1 g/mol), abnormal levels in the blood may be associated with illness and disease, thus it is crucial to be able to monitor them. Unfortunately, they all have similar structures with the same hydroxyl groups spatially arranged only slightly differently as the epitopes. This makes it difficult not only to select an aptamer that binds well but also that binds with high specificity. Organic or organometallic receptors were initially incorporated with aptamers to help resolve this issue and promote the interaction of the aptamer with the target, however the receptors instead interfered with binding. Nevertheless, Yang et al. (2014) used the receptors for the selection of aptamers for the aforementioned monosaccharides but in a different manner. Rather than combining the receptor with the aptamer, the receptor, specifically Shinkai's glucose fluorescent receptor, was first combined with the sugars to form a complex then added to an oligonucleotide pool for selection. The library was bound to an agarose streptavidin column via a biotinylated complementary oligonucleotide and the receptor and target, which already formed a complex, were added to the mixture. Sequences that bound the complex, having formed a ternary complex, dissociated from the complement on the column. Following counter-selections against the receptor and the selection of high affinity aptamers, a sensor was easily established due to the structure–switching selection method. In this method, the sensor is composed of a fluorescently-labeled aptamer bound to a quencher-labeled DNA strand. Upon addition of the target-receptor complex, the aptamer dissociates from the quencher strand and binds the complex, thereby restoring fluorescence of the aptamer and acting as a visible signal for the presence of target. The aptamers for each monosaccharide had high specificity as a result of the counter-selections that were performed against the receptor alone and the other sugars with the receptor. Furthermore, counter-selections were run against a mixture of steroids to remove any hydrophobic three-way junctions which are known to dominate selection experiments (Kato et al., 2000; Laing and Juliano, 2015). These methods were found to be successful in the selection of aptamers for monosaccharides that lack suitable epitopes for aptamer binding. This technique could be applicable for other small molecules which do not contain suitable functional groups for binding of aptamers during selection.

These are only a few methods from the past few years where researchers have modified SELEX and oligonucleotide structure to better select aptamers for small molecule targets. Although some limitations are still present, there have been considerable contributions to overcoming certain challenges that hinder SELEX for small molecules. They allow for better selections, thus yielding high affinity binders. Nonetheless, there is always room for improvement and more research.

Presented here is a list of aptamers for small molecules with their associated K_d_ that have been selected in the past 3 years (Table 1). A comprehensive list of aptamers for small molecules before and including 2012 can be found in the review by McKeague and DeRosa (2012).

**Table 1:** **Aptamers selected for small molecules from 2012 to 2015 and their respective K_***d***_ or limit of detection (LOD)**.

**Aptamer Target**	**RNA/DNA**	**K_*d*_ or LOD**	**References**
(Z)-4-(3,5-difluoro-4-hydroxybenzylidene)-1,2-dimethyl-1H-imidazol-5(4H)-one (DFHBI)	RNA	360 nM	Filonov et al., 2014
17β-estradiol	DNA	0.9 μM	Vanschoenbeek et al., 2015
17β-estradiol	DNA	50 nM	Alsager et al., 2014
Aflatoxin B1	DNA	11.39 ± 1.27 nM	Ma et al., 2014
Aflatoxin B2	DNA	9.83 ± 0.99 nM	Ma et al., 2015
Aflatoxin B1	DNA	96.6 ± 8.6 nM	Malhotra et al., 2014
Aflatoxin M1		35.6 ± 2.9 nM	
Anatoxin-A	DNA	81.3 ± 8 nM	Elshafey et al., 2015
Atrazine	DNA	0.62 ± 0.21 nM	Williams et al., 2014a
Benzylguanine	RNA	219.1 ± 7.8 nM	Xu et al., 2016
Brevetoxin-2	DNA	42 nM	Eissa et al., 2015
Bromacil	DNA	9.6 ± 7.8 nM	Williams et al., 2014b
Cd (II)	DNA	34.5 nM	Wu et al., 2014
Danfloxacin	RNA	2.99 ± 2.12 nM	Han et al., 2014
Fumonisin B1	DNA	62 ± 5 nM	Chen et al., 2014a
Kanamycin A	DNA	3.9 μM	Stoltenburg et al., 2012
Kanamycin A	DNA	2.8 μM	Nikolaus and Strehlitz, 2014
Ketamine	DNA	0.59 μM	Sun et al., 2014
Lysergamine	DNA	73 nM	Rouah-Martin et al., 2012
Malathion	DNA	1.14 ± 0.7nM	Williams et al., 2014c
Methamphetamine	DNA	100.2 ± 16.9 nM	Ebrahimi et al., 2013
N-methyl mesoporphyrin (NMM)	DNA	0.88 ± 0.12 μM	Yang and Bowser, 2013
Ochratoxin A	DNA	110 ± 50 nM	McKeague et al., 2014
Okadaic Acid	DNA	77 nM	Eissa et al., 2013
Okadaic acid	DNA	1.05 nM	Lin et al., 2015
Organophosphorus pesticides	DNA	Phorate: 1.11 μM	Wang et al., 2012
		Profenofos: 1 μM	
		Isocarbophos: 0.83 μM	
		Omethoate: 2 μM	
Oxytetracycline	DNA	4.7 nM	Kim et al., 2014
Pd II	DNA	4.60 ± 1.17 μM	Cho et al., 2015
Polychlorinated biphenyls	DNA	4.02 ± 0.54 μM	Xu et al., 2012
Polychlorinated biphenyls	DNA	66.36 ± 7 nM	Mehta et al., 2012
		99 ± 12 nM	
Progesterone	DNA	9.63 ± 3.12 nM	Contreras Jiménez et al., 2015
T-2 toxin	DNA	20.8 ± 3.1 nM	Chen et al., 2014b
Tebuconazole, inabenfide, and mefenacet	DNA	Inabenfide: 191 nM (LOD)	Nguyen et al., 2014
		tebuconazole 128 nM (LOD)	
		mefenacet: 276 nM (LOD)	
Tobramycin	DNA	150 nM	Spiga et al., 2015
Zearalenone	DNA	29 ± 5 nM	Chen et al., 2013

*Only the aptamer with the lowest K_d_ or limit of detection is reported*.

## Characterization of small-molecule binders

Screening and characterizing aptamers for small molecules is difficult; there are no universally accepted guidelines, making it challenging to choose the right assay for accurate affinity measurements. In addition, aptamers are considerably larger than small molecules, which can lead to high signal/noise ratio in size-based measurements, thus only sensitive assays will be able to detect their interaction. Current assays which have been demonstrated to effectively measure aptamer-target interactions include surface plasmon resonance (SPR), isothermal titration calorimetry (ITC), fluorescence polarization/anisotropy (FA/FP), and capillary electrophoresis (CE), however each assay has its own set of limitations and not all are suitable depending on the downstream application of the aptamer.

In this section, descriptions of innovative methods which overcome some of the current limitations are presented.

To begin, McKeague et al. (2015a) have tested and compared a variety of affinity assays, in an effort to streamline the workflow for screening and characterizing the binding of aptamers to small molecules. For their experiments, they chose the small molecule ochratoxin A (OTA, M_W_: 403.8 g/mol), a food-contaminating fungal toxin, and multiple previously reported OTA aptamers. The use of different aptamers for the same target allowed for comparisons amongst them as well. The aptamers were put through a panel of binding assays, including equilibrium dialysis, ultrafiltration, affinity chromatography with magnetic beads, fluorescence polarization, SPR (Chang et al., 2014), a modified DNase I protection assay (Frost et al., 2015), a SYBR green displacement assay (SG), and a gold nanoparticle protection assay (AuNP). Notably, no one aptamer performed equally well over all the different assays; authors reported inconsistencies in the binding affinities (K_d_s) between the different assays when the same aptamer was used. Furthermore, although all aptamers displayed binding to OTA in at least one assay, the strength of their interaction widely varied across the different assays, with the binding of several aptamers with certain assays not detectable. As a result, it was deemed that it would be favorable to use multiple assays when characterizing small molecule aptamers, in order to overcome any inherent limitations of individual characterization approaches. Some assay limitations include immobilization of either the aptamer or the target, use of labeling, cost, lack of sensitivity inability to measure binding quantitatively, or the involvement of complicated surface chemistry. As an example, the magnetic bead test involves target immobilization, which can create steric hindrance and impair binding due to the blocking of an original exposed site on the target. Thus, performing an additional test in which the target and aptamer are free in solution and thereby allowing free movement, for example the DNase I protection assay (see below) allows for a fuller assessment of the functionality of the aptamer. Ultimately, due to limitations in each assay, inconsistencies in K_d_s between different assays, and to confirm the functionality of the aptamer, it is highly recommended to select assay conditions which mimic the desired application of that aptamer. In this way, by not drastically changing binding conditions, the measured binding affinity becomes a truer indication of how the aptamer will perform in the downstream sensor, or other application.

To improve aptamer development, McKeague et al. designed a workflow which comprises the methods, assay considerations, and assay options for aptamer screening, characterization, and functional verification once the SELEX process is complete and several aptamer candidates have been revealed by sequencing. The workflow sets out suggestions for appropriate combinations of assays for use in (1) the initial screening of aptamer sequenced, (2) truncation of aptamers, (3) K_d_ characterization, and (4) functional validation. For example, screening methods such as FP, SG, AuNP, and affinity chromatography with magnetic beads were recommended for their dependability, rapidity, and cost effectiveness. By following this workflow diagram, the screening can be done efficiently, the aptamer affinity can be quantified, and one can have some confidence that the chosen aptamers will perform well in the downstream application.

Despite the inherent challenges with the characterization of small molecule-binding aptamers, recently, there have been several promising advances. In particular, characterization methods which have been proven effective for characterizing aptamer binding to larger molecules such as proteins have been adapted in innovative ways to suit smaller targets.

A notable advance for screening aptamers for small molecules was made with the use of a nanoporous composite formed by the sol-gel process. This process will allow for the screening of aptamers for low molecular weights targets, suitable for the first step in the aforementioned workflow diagram. The technique of sol-gel entrapment is compatible with biological molecules such as aptamers due to its aqueous environment which allows for the aptamers to function optimally. The silica composite employed in this method is made up of many pores of adjustable size to trap the small molecule target. Silica composites with pores large enough to trap proteins have already been studied (Kim et al., 2006), and was recently adapted to work with small molecule bisphenol A (M_W_: 228.3 g/mol), a potentially harmful industrial chemical, by Ahn et al. (2012), with a few important modifications. The available size range of the pores in the original approach was limited as pore sizes below a certain threshold compromised the adhesiveness of the sol-gel matrix to commonly used surfaces, for example microtiter plates, for automatization. Therefore, in order to access pores in the size range that would be suitable for small molecule entrapment, a new porous silicon substrate was generated and which also allows the sol-gel composite to attach to any surface. With this innovation, many surfaces and types of sol-gel solvents can be used. The aptamer has been shown to freely move within the complex morphology of the nanostructural composite and reach the target, and the conventional recovery methods (i.e., heat) of the aptamers bound to the target can also be used, such that the aptamers can be quantified or amplified using PCR. This method does not require the immobilization of either the target or the aptamer, thereby allowing for free binding. Also, this method could potentially be used for the selection of aptamers for small molecules (SELEX) to overcome the issue of attaching either the target or the DNA pool to a solid support matrix, which could potentially impact the binding of the aptamer to the target. In addition, this method can be automated since the porous silicon sol-gel surface can be used on standard microtiter plate formats. The labeling of aptamer with a fluorescent probe required for monitoring is the main weakness of this method. The probe could potentially affect the folding of the DNA which would impact the binding to the target, besides adding to fabrication costs.

**Figure 3:**
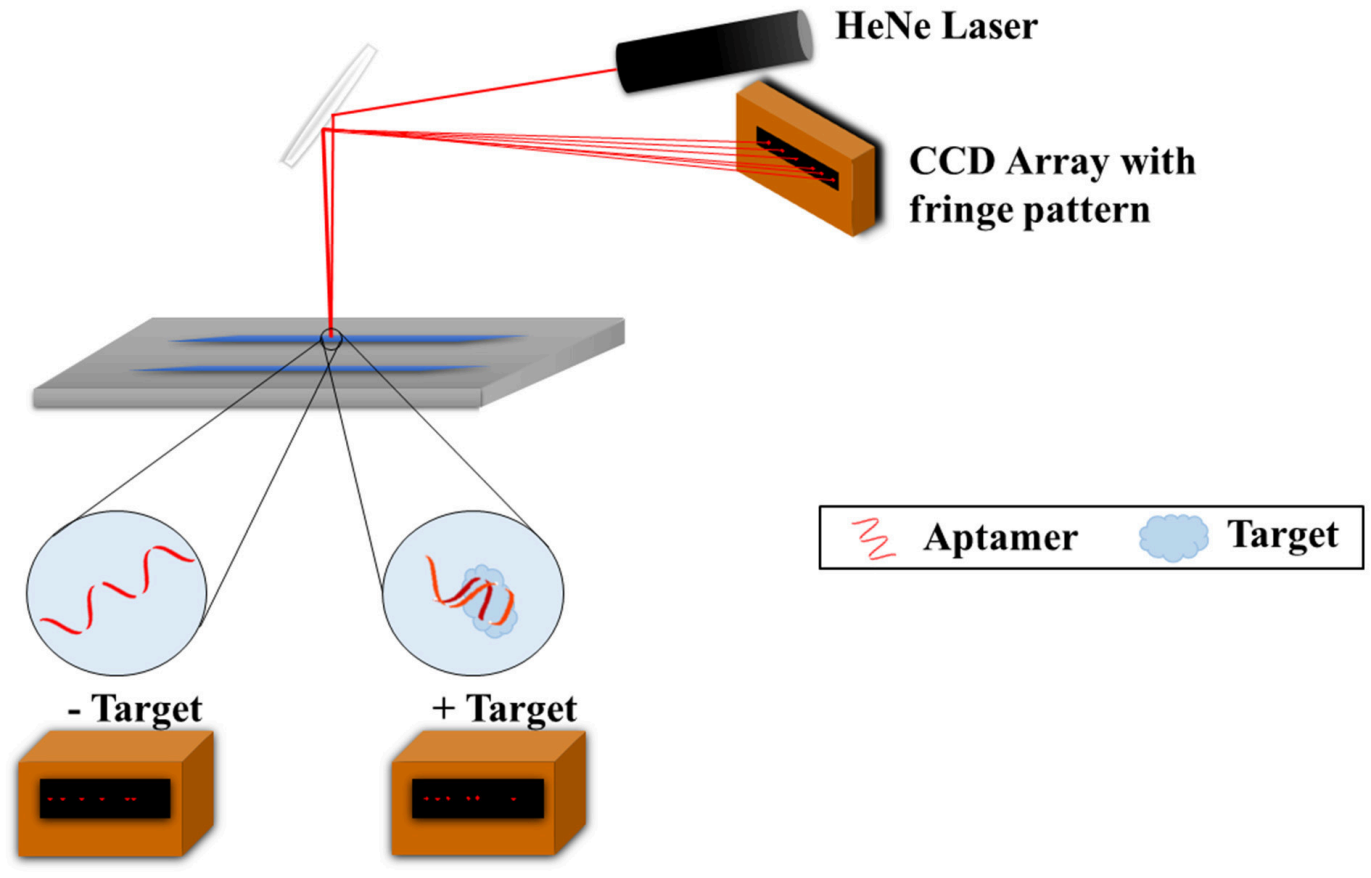
**Backscattering interferometry setup for aptamer-target binding measurements**. The target and aptamer are incubated together in a microfluidic channel. A helium-nexon laser irradiates the sample and the beam is reflected and refracted within the channel. The laser exits as a fringe pattern and is detected with the CCD array. Binding of the aptamer to the target changes the refractive index of the laser and subsequently the fringe pattern, and as such binding can be studied.

Another noteworthy method for characterizing aptamers for small molecules is with the use of backscattering interferometry (BSI). This method was successfully applied for measuring aptamer affinity to larger molecules such as proteins but Kammer et al. (2014) have succeeded with small molecule antibiotics, specifically tenofovir, epirubicin, ampicillin, tetracycline, and the hormone/neurotransmitter norepinephrine (M_W_: 287.2, 543.5, 349.4, 444.4, 169.1 g/mol, respectively). BSI involves the use of a microfluidic chip, a helium-neon laser, and a charge coupled device (CCD) array (Figure 3). A channel is etched in the chip which functions as the optics and a sample holder. The laser irradiates the sample, the beam is reflected and refracted within the channel, and exits as a fringe pattern before being detected with the CCD array. Binding of the aptamer to the target leads to a refractive index change which results in changes to the fringes' position, thus allowing for the studying of the binding. Both the aptamer and target are free in solution and do not require any labeling; this is possible due to the fact that the BSI signal is based on the target conformation, dipole moment, and hydration. This method was shown to produce quantitative K_d_ values which matched very well with the K_d_s obtained from SPR published in the literature.

An interesting method for characterizing the binding of aptamers and small molecules, and determining the binding site on the aptamer, is with the use of MicroScale Thermophoresis (MST). Entzian and Schubert (2015) performed this method with adenosine triphosphate (ATP, M_W_: 507.1 g/mol) aptamers. The study of how the movements of molecules change over temperature gradients (hence thermophoresis) is the principle of MST. The movement in the temperature gradient is dependent on size, charge, and hydration shell of the molecule. When the target is introduced and binds to the aptamer, one or more of those properties changes and alters its movement. The thermophoresis movement is recorded in thin glass capillaries, where microscale sized temperature gradients from 2 to 6°C are induced by an infrared laser. By focusing on other parameters in addition to mass, targets of any size can be used and as a result, the interaction with the aptamer can be measured. This method can measure affinities in the pM and mM range, which is suitable since the affinities of aptamers for small molecules are generally nM or μM. This method offers affinity measurements in real-time and in under 15 min and can be performed in complex media, although at times labeling is required.

The researchers have taken this method a step further and aimed to determine the exact binding site of the target. Strategically, they used structural analogs of ATP and exposed them to the ATP aptamer. It was found that the binding affinities of the aptamer to the ligands varied widely; some ligands displaying low binding and others displaying no binding at all. By relating the binding information to the ligand structures, areas of the structure that had major and minor influences on binding could be elucidated.

The DNase I footprinting assay is a conventional characterization assay that can be employed for the study of aptamer-target binding interactions. Briefly, the binding of a target molecule to an aptamer could lead to regions within the oligonucleotide structure that would be protected from digestion; thus, differences in digestion patterns in the presence and absence of target are correlated to binding interactions. This method was previously only used with large molecule targets since these changes are more easily observed with larger targets, until Frost et al. (2015) executed the assay with the aptamer for small molecule fumonisin B1(M_W_: 721.8 g/mol); fumonisin B1 is a toxic fungal metabolite that that contaminates maize. This assay involves target and 5′-fluorescently labeled aptamer free in solution. Following incubation of the aptamer and increasing concentrations of target, the enzyme DNase I is added and partially digests the aptamer since the enzyme highly prefers cleaving double stranded DNA over single stranded DNA. Only the cleaved 5′ segments with the fluorescent label are visible on a gel, where a footprint map is generated. By testing with various concentrations of target, a binding isotherm can be created to measure the affinity of the aptamer. Small changes in band intensities on the gel at different target concentrations are examined using image analysis to discern any structural perturbations that occur when the aptamer binds to the small molecule. Binding to the target results in structural changes in the aptamer where some areas previously double-stranded become single-stranded and are thereby digested, resulting in different fragments than when there is no binding. Thus, this map of fragments can indirectly provide structural and thermodynamic information at the site of the interaction. This method is a relatively quick and affordable assay for characterizing the binding of small molecules to aptamers. Unfortunately, due to the small subtle changes in band intensity, a larger error is associated with the K_d_. In addition, the aptamer must be labeled for monitoring on the gel. Nevertheless, this assay provides the researcher a method for measuring the interaction of an aptamer with a small molecule without the possible negative side effects involved in the immobilization of either molecule. It provides information on the structure of the aptamer upon target binding and can also be used as a quick aptamer screening method.

As previously mentioned, the issue of the drastic size difference between the aptamer and the small molecule is of concern. One way to overcome this problem is to truncate the aptamer as much as possible while still retaining the maximum binding. Researchers Frost et al. (2015) used the DNase I assay to accomplish this. Not only is this method suitable for determining the binding affinities of potential aptamer minimers, this method infers which minimers to focus on; fluorescent bands visible on the gel following digestion of the full aptamer are fragments. The bands with consistent changes in intensity relative to target concentration are fragments likely directly involved in the binding interaction with the target. The fragment size can be determined by measuring the distance traveled on the gel, and the section of the aptamer that size corresponds to that fragment size can be determined using programs which predict the secondary structure. As a result, any structures (ex. stem loops) involved in the binding can also be determined. Alternatively, if digested bands do not show consistent change in band intensity relative to target concentration, then binding is not occurring.

Another way for reducing aptamer size while maintaining high binding to the target is the bioinformatics approach as done by researchers Sánchez-Luque et al. (2014). Although the target in this case is not a small molecule, it is still applicable and can be beneficial for small molecule aptamers. The researchers used algorithms to fold sequences into their most stable secondary structure, make predictions on structural position, determine the preferred site of interactions between the sequence and the target, and more. The collection of information assists in the determination of minimer aptamer sequences.

Both the DNase I assay and the bioinformatics approach have yielded truncated aptamers that maintained maximum binding.

The above-mentioned studies provide structure and alternatives to conventional methods of characterizing aptamer-small molecule interactions. Reliable and efficient aptamer screening methods are vital to avoid excessive costs and time by testing many sequences. As for characterization, it is worthwhile to test the K_d_ using multiple affinity methods to ensure a precise K_d_ is obtained. In addition, the method chosen should match the application in which the aptamer would be used for, such that the K_d_ from the test accurately reflects the K_d_ in the application.

## Detection using small-molecule binders

Sensitive, affordable, and easy to use detection devices for small molecules such as toxins, antibiotics, molecular markers, drugs, and heavy metals are in demand by various industries, including health care, agriculture, environment, and forensics. This need is ultimately for the protection and wellbeing of humans and animals.

A biosensor, a small analytical device involving a biological molecular recognition element with a physiochemical transducer (Sefah et al., 2009), can qualitatively or quantitatively detect a small molecule of interest. The biological component interacts with a target, and the transducer component converts that interaction into a measurable signal. The biological component can be made up of proteins, antibodies, or aptamers and the success of the sensor is based on the affinity and selectively of the chosen recognition element. A biosensor that utilizes aptamers as the recognition element is called an aptasensor. While sensors are analytical devices, assays are analytical procedures that would measure quantitatively or qualitatively the small molecule of interest.

Currently, antibodies are the most popular biological components for biosensors and assays, greatly overshadowing other options. Nonetheless, aptamers are slowly making their way into the mainstream because they are robust and reusable, unlike antibody-based sensors, and their small size means there can be high density immobilization on the sensing surface.

As previously discussed, when it comes to working with aptamers and small molecules, the large size difference between the two is an impediment which can make designing a detection assay sometimes difficult. For example, it was found via NMR analysis of aptamer-argininamide interactions that, argininamide (M_W_: 173.2 g/mol) is sandwiched between reversed Hoogsteen and Watson-Crick base pairs and tucked into the aptamer binding area as if it were a cavity (Lin and Patel, 1996). This type of encapsulation suggests that the small molecule might be masked from other probes (Hermann and Patel, 2000). As such, assays for small molecule detection are generally single-site binding configurations, which make sandwich assays with a secondary aptamer challenging.

Current detection methods for small molecules are often spectroscopy-based, such as fluorescence polarization, and UV absorption. There are also separation-based methods such as HPLC, equilibrium dialysis, affinity chromatography, and other methods such as thin-layer chromatography (TLC) and isothermal titration calorimetry (ITC; for a more comprehensive review please refer to McKeague and DeRosa, 2012). However, these methods either require labeling, the use of expensive machinery and equipment, are labor intensive, time consuming, or experience non-specific binding. In some methods, more than one limitation exists.

To overcome some of these limitations and to increase the sensitivity of aptamer-based detection systems for small molecules, researchers have developed new proof-of-concept methods or altered longstanding methods. Notable modern advances, post 2012, are described.

**Figure 4:**
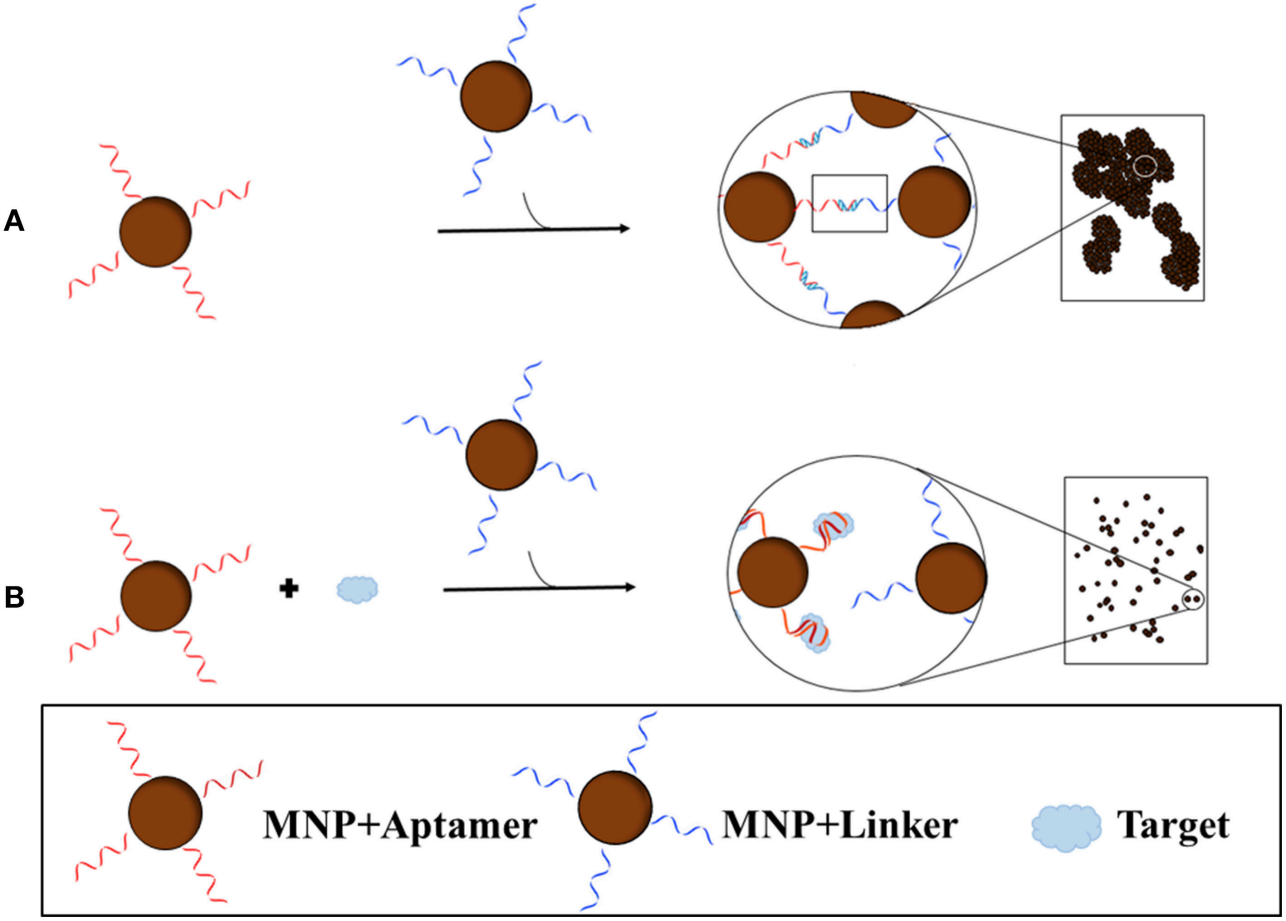
**Blu-ray aptasensor scheme for the detection of target**. Two types of MNPs are necessary; one MNP contains an aptamer covalently bound while the other contains a short complementary sequence of the aptamer. In the absence of target, the two MNPs form clusters as a result of the binding of the aptamer to the complementary sequence **(A)**. In the presence of target, the MNP with the aptamer will preferentially bind the target than the complement on the other MNP and thus few or no clusters will form **(B)**. Cluster density can then be measured by the Blu-ray unit.

Recently, Yang et al. (2015) developed a blu-ray aptasensor for the detection of ATP. This method utilizes a blu-ray optical pickup unit and two types of magnetic nanoparticles (MNPs); the first MNP contains the ATP aptamer covalently bound while the other contains a short complementary sequence of the aptamer (Figure 4). Both MNPs are incubated together, forming MNP clusters as a result of the binding of the aptamer to the complementary sequence. Once the target is introduced, the aptamer, which has a higher affinity for the target than the complement, will bind to the target and dissociate itself from the complement. The binding of the aptamer to the target inhibits MNP clustering; therefore the amount of clustering is inversely proportional to the concentration of target. To quantitatively measure the cluster density, transmitted light is collected from the blu-ray optical pickup unit, and since the optical signal is related to the rotational dynamics of the MNPs, a measure of the hydrodynamic size distribution of the clusters can be obtained. The concentration of ATP was determined by quantitatively measuring the clustering of the MNPs; the optomagnetic reading of the cluster size correlates to the concentration of ATP, allowing for accurate determination of the target concentration. Being very inexpensive and a small, compact machine, this device has the potential to become a portable sensor.

A popular method for studying molecular interactions and detecting the presence of a target is fluorescence anisotropy. Fluorescence anisotropy is a solution-based method, thereby allowing free movement of the aptamer and target. With this technique, a biomolecule must be fluorescently labeled to monitor the interaction. Polarized light of a specific orientation is used to excite the fluorophore which then emits light. Only the light emitted from the molecule with the same orientation is detected and an anisotropy signal is obtained. High or low anisotropy signal is dependent on the size of the fluorescent molecule. In the case of an aptamer and small molecule target, a fluorescent aptamer will rotate and tumble quickly, thus the emitted light is not conserved long and yielding low signal. However, when bound to a protein, the aptamer becomes a large complex which tumbles slowly, conserving the emitting light, and thus yielding a large signal. Thus, based on mass changes of the fluorophore, this method gives information on the orientation, mobility, and interaction of biomolecules.

This method is not always practical with smaller targets as their small size would not result in a substantial difference in anisotropy signal. Signal amplification would be necessary to modify this method to suit small molecules as explained by researchers Liu et al. (2013) who performed fluorescence anisotropy with graphene oxide (GO) for ATP (M_W_: 507.1 g/mol) detection. GO preferentially binds ssDNA and acts as a scaffold, and also a quencher of fluorophores due to Förster resonance energy transfer between fluorescein and graphene. By applying these properties, two versions of FA were executed: (1) A fluorescently labeled aptamer that binds to GO in the absence of target results in decrease in fluorescence due to quenching and a size increase resulting in high anisotropy signal. Once in the presence of target, the aptamer will preferentially bind to the target, restoring its fluorescence and forming a complex which is smaller in size, resulting in a low anisotropy signal; (2) A duplex formed between a fluorescent DNA probe whose sequence is complementary to an unlabeled aptamer does not bind to GO in the absence of target, therefore fluorescence is visible and anisotropy signal is low. In the presence of target, the aptamer dissociates from the fluorescent probe to bind the target and the probe binds to GO. The probe and GO form a large complex resulting in a high anisotropy signal but the fluorescence is quenched. Since the probe is the labeled molecule, the interaction of the aptamer and target is measured indirectly. Regardless, the difference in signal with and without target in both versions was found to sufficiently large enough to discern binding. This method which incorporates GO as a signal amplifier successfully detected ATP in a rapid fashion, requiring seconds to obtain a signal and only minutes to reach equilibrium. This method, which does not rely on a conformational change for the aptamer has also shown to be highly specific to ATP, and has been shown to function in serum as well as buffer.

Oligonucleotide-based sensors with one-dimensional nanostructures are also used for the sensitive detection of small molecules. For example, Gong et al. (2013) generated a sensor for the detection of Hg^2+^ (M_W_: 200.6 g/mol) using single-walled carbon nanotubes (SWNTs). The methodology of this sensor is based on an older report from Das et al. (2011) which used a SWNTs-based chemiresistor to detect ATP (M_W_: 507.1 g/mol) with picomolar sensitivity and high selectivity. In the 2013 study an oligonucleotide PolyT strand has affinity for Hg^2+^. A free PolyA strand is hybridized with the PolyT functionalized SWNTs. The PolyT strand acts as the detection probe, as the PolyT will structure-switch upon binding Hg^2+^ preferentially over the PolyA, resulting in the release of PolyA from the surface of the SWNT. Hg^2+^ is sensed by the increase in source and drain current (or the decrease in resistance) due to the release of PolyA. This label-free sensor was found to have high sensitivity, in addition to high selectivity. Compared to other ions (Ca^2+^, Mg^2+^, Mn^2+^), a much greater decrease in resistance was observed with Hg^2+^, thereby confirming the high selectivity to Hg^2+^.

Methods such as QCM are generally not performed with small molecules because their molecular mass is too small and do not cause a measurable size shift when the aptamer is bound compared to when aptamer is not bound. With large molecule targets like proteins, these methods are sensitive and reliable. Being able to benefit from such sensitive tests would be valuable, thus researchers have recently taken on the challenge of applying this method successfully for small molecules. The following methods of using QCM are not only highly sensitive, but allow for direct detection of binding in real-time, without the need of labeling or signal amplification, and can detect minute changes in mass densities. Özalp (2011), followed by Osypova et al. (2015), have successfully used QCM coupled with dissipation monitoring (QCM-D) for the detection of small molecules ATP (M_W_: 507.1 g/mol) and a mimic of the amino acid tyrosine, L-Tyrosinamide (M_W_: 180.0 g/mol), respectively. The dissipation monitoring allows for increased sensitivity as it monitors changes in resonance frequency and variations in energy dissipation upon target binding to aptamer films, properties from which mass differences can be calculated. Özalp performed the method by initially immobilizing biotin-labeled aptamers via avidin onto a gold surface. A decrease in frequency and an increase in dissipation energy were observed when target was added. With frequency and dissipation data, and a viscoelastic representation of the film, small mass changes on the surface were resolved. This method was able to detect ATP at low concentrations, is a rapid technique, and has been shown to work perform well in serum as well as buffers.

The method of Osypova et al. is quite similar to that of Özalp although it takes advantage of the change in conformation of the aptamer upon target binding and the effect that has on hydration of the sensing layer. By using a QCM-D with spectroscopic ellipsometry the thickness, refractive index, optical mass, acoustic mass, and hydration of the aptamer layer can be measured all at once. QCM-D measures the mass uptake of the target to the aptamer but also includes the mass of water hydrodynamically bound to the aptamer. Upon target binding to the immobilized aptamer, the aptamer undergoes a conformational modification, which decreases the aptamer monolayer thickness resulting in the release of measurable water to which it was coupled. QCM-D can therefore detect the release of water. By taking into account the mass of water, this method becomes more sensitive than optical techniques that rely on refractive index measurements that only measure the mass of the adsorbed molecule. As a result, the method becomes sensitive enough for detection of small molecules. On a side note, although this method is sensitive and can detect small molecules with masses below 200 g/mol, it requires aptamers that change conformation upon binding with the target for successful detection.

Similarly, another interesting method for detecting small molecules is a proof-of-concept work with an electromagnetic piezoelectric acoustic sensor (EMPAS). Neves et al. (2015) performed this method for the detection of cocaine (M_W_: 303.3 g/mol). The aptamer was immobilized onto an electrode-free piezoelectric surface, namely quartz substrate sensing platform, via a linker known as S-(11-tri- chlorosilyl-undecanyl)-benzenethiosulfonate. To minimize steric hindrance due to tightly packed aptamers, MCH was added to react with the linker and provide spacing. This spacing would allow for better binding of the target to the aptamer. The excitation of the acoustic resonance due to an external magnetic field produced by a spiral coil 30 μm underneath the quartz substrate occurs at a distance, which allows the sensor to function at ultra-high frequencies (>1 GHz). The distant excitation is what makes this method unique from other piezoelectric sensors. The wide frequency range allows for tweaking the signal-to-noise ratio, thereby increasing sensitivity of the sensor. The acoustic wave device allows for measurement of direct binding of the aptamer and the target by changes in mass difference and without any labeling required. Additionally, this proof-of-concept method uses an aptamer which only changes its tertiary structure upon target binding. Its secondary structure is already folded before the target, and there are no considerable changes in secondary structure upon binding, thereby indicating that this method does not rely on structure switching in order to perform well, as the case with other sensors. In this method, the aptamer bound cocaine with an affinity comparable to what has been detected using other techniques. While the sensor is not as sensitive as others that use the current cocaine aptamer, this method is a one-step process that does not require signal amplification, labeling, or aptamer structure-switching. When compared to methods that do not amplify signals, this method fares comparably. This method can also be regenerated for continuous use.

Finally, SPR can be challenging to adapt to small molecule detection because it is a mass-sensitive technique. Normally, SPR is used with large molecules that result in large changes in refractive index induced by binding to the aptamer on the surface. Nonetheless, Park et al. (2014) were able to apply it to small molecules using localized SPR (LSPR) for the detection OTA. Here, label-free LSPR is used to detect the binding of the target to aptamers immobilized on the surface of gold nanorods. Again, this method takes advantage of a conformational change in structure of the aptamer upon target binding, thus the aptamer of choice self-assembles into a G-quadruplex upon interaction with OTA. The geometric change associated with formation of the G-quadruplex induces a red shift of the LSPR peak as a consequence of a refractive index change occurring at the gold nanorod surface. The change in shape upon target interaction increases the density of the surface and decreases the thickness which, in combination the stability provided by having the aptamer close to the surface of the nanoparticle, leads to augmented LSPR wavelength shift and ultimately a more sensitive sensor. A linear response was found for LSPR redshifts as a function of OTA concentrations. Not only is this sensor sensitive, it can be regenerated for re-use over 7 times. This method label-free and also functions well in corn samples spiked with OTA.

These recent advances in aptasensor development for the detection of small molecules are promising, with some having overcome aforementioned obstacles such as the aptamer and target mass difference, the need to label the aptamer, expensive machinery, and time-consuming methods. While the progression is encouraging, more effort is needed for easier to use sensors that avoid immobilization of the aptamer and target to allow free equilibrium binding.

## Conclusion

Recent advances have contributed to easier, quicker, and more reliable methods for the selection, characterization, and application of aptamers for small molecules. By modifying SELEX and oligonucleotide structure, better selections have been designed to produce aptamers that bind small molecules targets with high affinity and specificity. Recent methods of screening aptamers and characterizing their interactions with targets have provided structure and alternatives to conventional methods, along with the understanding that the method of characterization chosen should match the application in which the aptamer would be used for. This way the K_d_ from the test accurately reflects the K_d_ in the application. Finally, approaches for aptamer-based biosensors have overcome some aforementioned obstacles such as the aptamer and target mass difference, the need to label the aptamer, expensive machinery, and time-consuming tests. The immobilization of the small target or oligonucleotide remains one of the biggest impediments in developing an aptamer for small molecules as free equilibrium binding is unachievable. Nonetheless, many advancements are being made, and more are likely to be made due to the ever-increasing popularity of aptamers and the persistent need for detection of small molecules in various industries for the protection of humans and animals.

## Author contributions

AR wrote the manuscript and prepared the figures. MD wrote and edited the manuscript.

## Funding

The authors wish to acknowledge the Natural Sciences and Engineering Council of Canada for a graduate scholarship to AR and Discovery Grant to MD.

### Conflict of interest statement

The authors declare that the research was conducted in the absence of any commercial or financial relationships that could be construed as a potential conflict of interest.
